# Supplementary material for: Tightly Knotted Enzymes Inhibit Protein-Protein Aggregation
Source: bioRxiv. 2026 Jun 13:2026.06.12.731949. Preprint. [Version 1] doi: 10.64898/2026.06.12.731949 (PMC13277964; doi:10.64898/2026.06.12.731949)
Supplement: Supplementary file 1 [file media-1.pdf]

Supporting Information for "Tightly Knotted Enzymes Inhibit Protein-Protein Aggregation"

Susmita Sarkar[1], Hemanth Mandya Nagaiah[1], Michael L. Klein[1], and Vincenzo Carnevale[1,*]

[1]Institute for Computational Molecular Science, Temple University, Philadelphia, Pennsylvania 19122, United States

*Corresponding author: vincenzo.carnevale@temple.edu

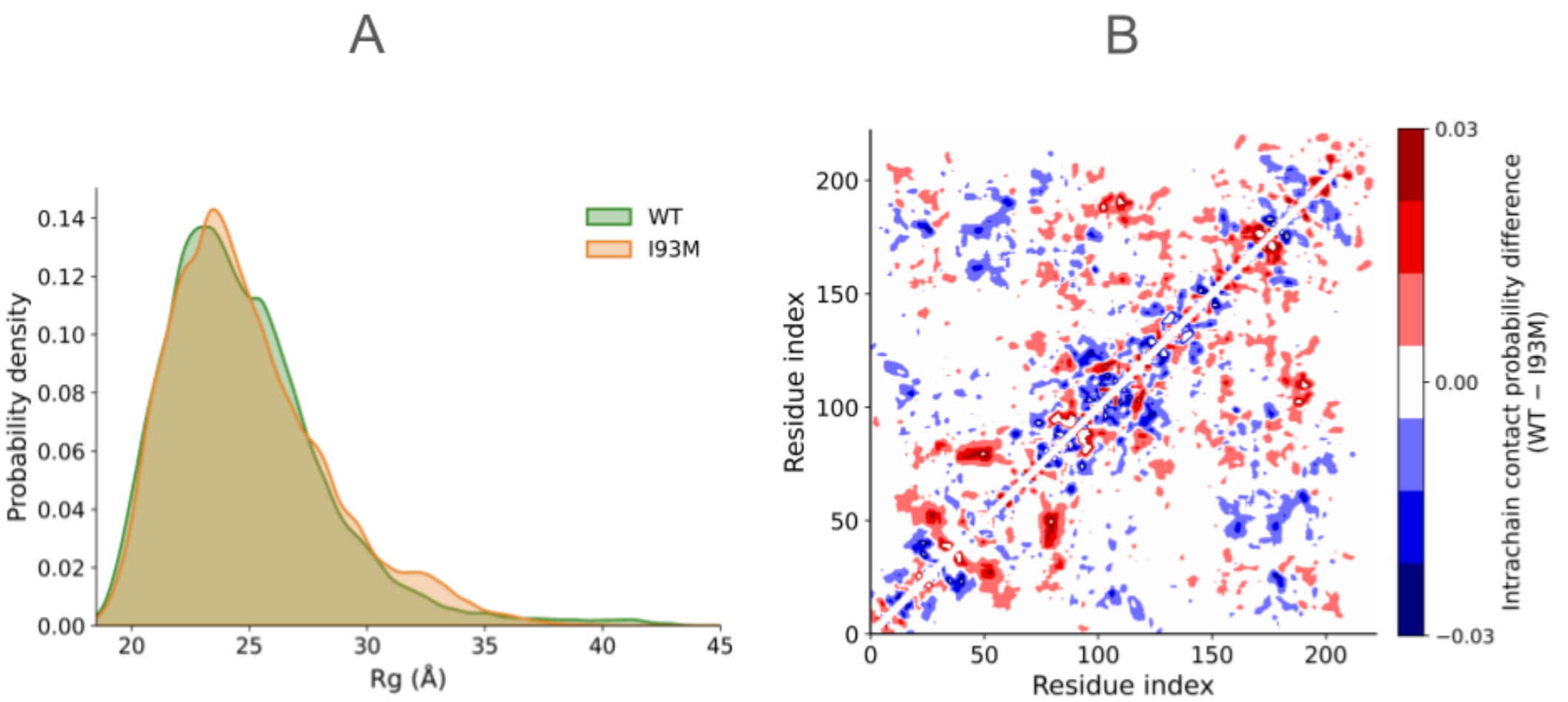

Figure S1: (A) R_g distributions of protein chains in the monomeric form in dilute phase from multichain simulations for WT and I93M. (B) Difference maps of intrachain contact probabilities between WT and I93M for protein chains in the monomeric form in dilute phase from multichain simulations, calculated as WT - I93M. The color scale (blue to red) indicates reduced (blue) and increased (red) contact probabilities in WT relative to I93M.

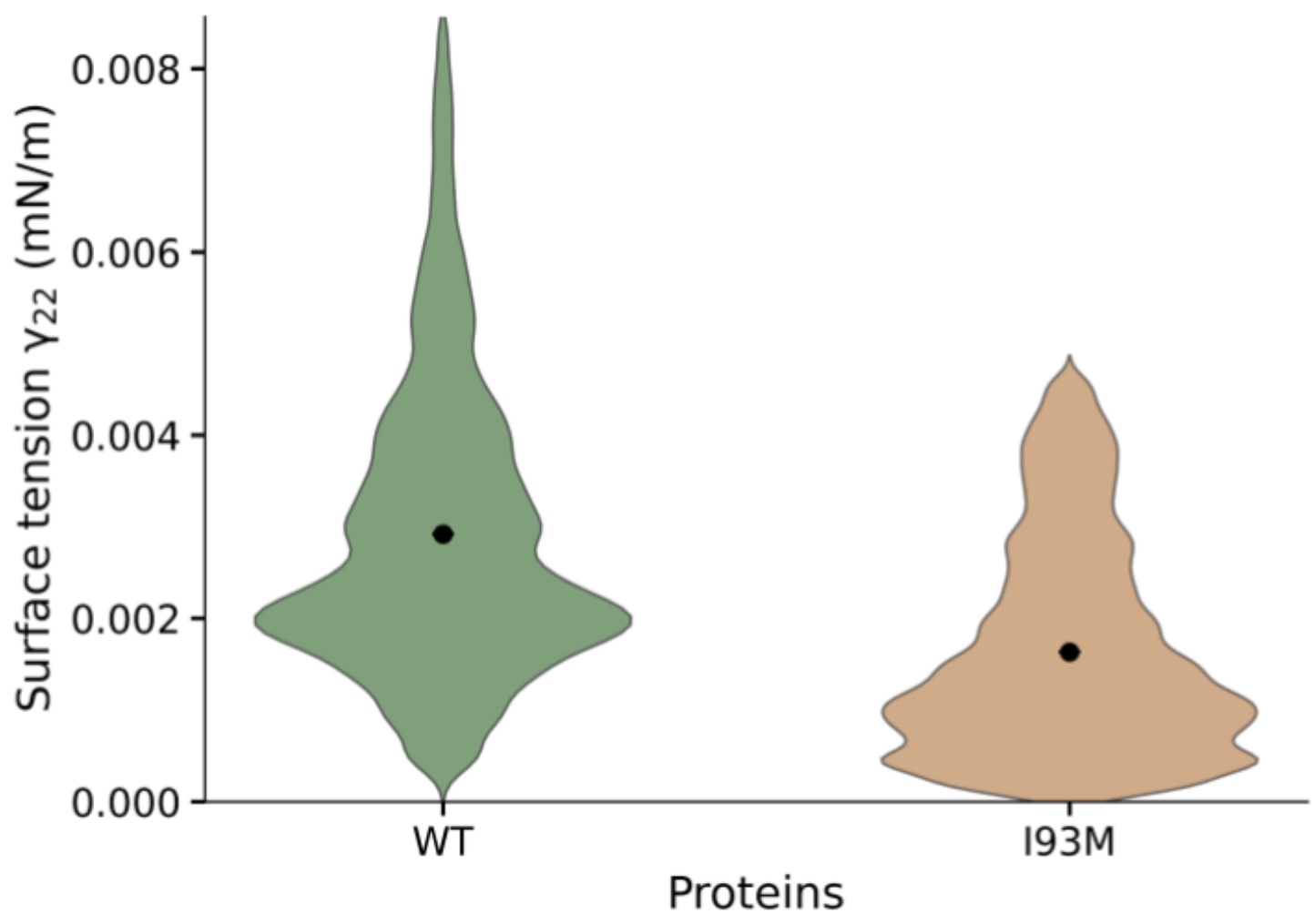

Figure S2: Surface tension (Γ_{22}) of condensates, indicating reduced mobility and increased resistance to flow in I93M.

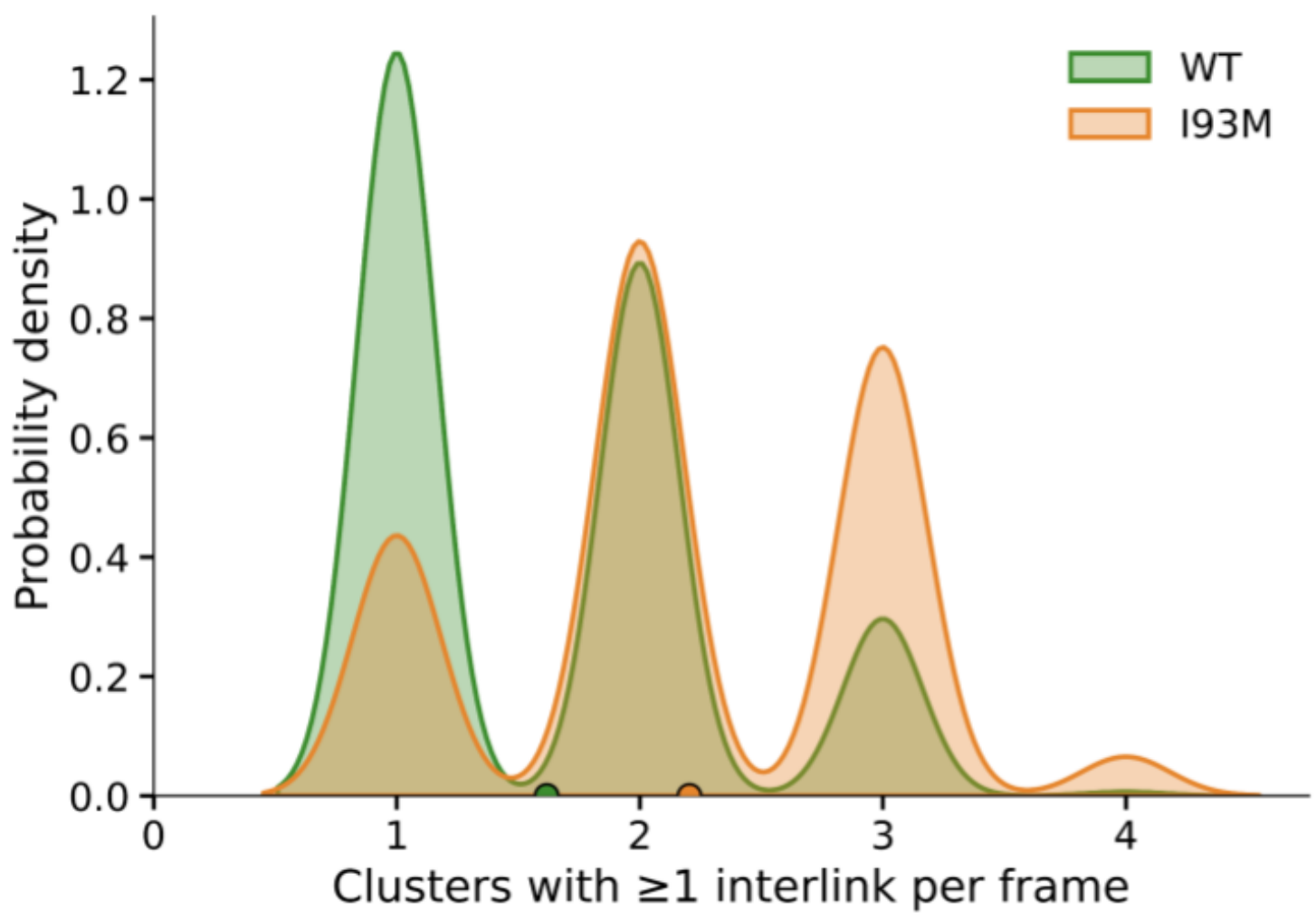

Figure S3: Probability distribution of the number of clusters with one or more than one interlinking per frame in WT and I93M.
